# Mechanisms underlying immunosuppression by regulatory cells

**DOI:** 10.3389/fimmu.2024.1328193

**Published:** 2024-02-06

**Authors:** Oliver Goldmann, Obiageli Vivian Nwofor, Qian Chen, Eva Medina

**Affiliations:** Infection Immunology Research Group, Helmholtz Centre for Infection Research, Braunschweig, Germany

**Keywords:** regulatory T cells, regulatory B cells, myeloid-derived suppressor cells, immunosuppressive mechanisms, infection

## Abstract

Regulatory cells, such as regulatory T cells (Tregs), regulatory B cells (Bregs), and myeloid-derived suppressor cells (MDSCs), play a crucial role in preserving immune tolerance and controlling immune responses during infections to prevent excessive immune activation. However, pathogens have developed strategies to hijack these regulatory cells to decrease the overall effectiveness of the immune response and persist within the host. Consequently, therapeutic targeting of these immunosuppressive mechanisms during infection can reinvigorate the immune response and improve the infection outcome. The suppressive mechanisms of regulatory cells are not only numerous but also redundant, reflecting the complexity of the regulatory network in modulating the immune responses. The context of the immune response, such as the type of pathogen or tissue involved, further influences the regulatory mechanisms involved. Examples of these immunosuppressive mechanisms include the production of inhibitory cytokines such as interleukin 10 (IL-10) and transforming growth factor beta (TGF-β) that inhibit the production of pro-inflammatory cytokines and dampen the activation and proliferation of effector T cells. In addition, regulatory cells utilize inhibitory receptors like cytotoxic T-lymphocyte-associated protein 4 (CTLA-4) and programmed cell death protein 1 (PD-1) to engage with their respective effector cells, thereby suppressing their function. An alternative approach involves the modulation of metabolic reprogramming in effector immune cells to limit their activation and proliferation. In this review, we provide an overview of the major mechanisms mediating the immunosuppressive effect of the different regulatory cell subsets in the context of infection.

## 1 Introduction

The immune system plays an essential role in host defense against pathogens. However, the immune response during infection needs to be properly regulated in order to effectively eliminate the infecting agent while also avoiding the detrimental effect of an excessive inflammatory reaction. Achieving this balance is important for the maintenance of immune homeostasis and preventing autoimmunity. For example, failure to control hyperinflammatory responses can lead to a cytokine storm that ultimately results in death (1). On the other hand, excessive dampening of the immune response poses a risk, potentially hindering the clearance of pathogens and contributing to the chronicity of infection (2, 3). In order to prevent both excessive responses and chronic infections, the immune system has evolved several mechanisms orchestrated by diverse subsets of regulatory cells for regulating the intensity and duration of immune reactions. However, because these regulatory mechanisms are mostly immunosuppressive, many pathogens have evolved strategies to hijack the regulatory mechanisms of the host for their own advantage, thus generating conditions that ensure their survival and persistence within the host. Therefore, in the context of infection, adjunctive therapeutic approaches that aim to ameliorate or modulate these suppressive mechanisms may be beneficial for improving the infection outcome.

The most prominent regulatory cell subsets include regulatory T cells (Tregs), regulatory B cells (Bregs), and myeloid-derived suppressor cells (MDSCs). Tregs are a specialized population of T cells that regulate the activity of CD4+ and CD8+ T cells as well as natural killer (NK) cells and are an essential component for the proper functioning of the immune system (4, 5). They play a pivotal role in preventing autoimmune diseases by dampening the responses of self-reactive lymphocytes (6, 7). Tregs are characterized by the expression of CD4 and the interleukin-2 receptor α-chain (IL-2Rα), commonly known as CD25 (4). A defining feature of Tregs is the expression of the forkhead box transcription factor Foxp3, a master regulator that plays a critical role in their development and function (8–10). Tregs also control the immune response to infectious pathogens, and in this context, their activity is not always beneficial (11). For example, Tregs can hinder the development of sterilizing immunity against specific pathogens by preventing an effective immune response (11, 12).

While B cells are typically recognized for their role in initiating a humoral immune response through the production of antigen-specific antibodies (13), a distinct subset called regulatory B cells (Breg cells) deviates from this conventional function and contributes to immune regulation (14–16). Whereas the regulatory function of Bregs is critical for the maintenance of immune balance, it can also benefit certain pathogens (17).

MDSCs are immature myeloid cells with vigorous immune-suppressive activity involved in suppression of effective immune responses in many pathological conditions, including cancer, chronic inflammation, autoimmunity, and infections (18, 19). Various pathogens, including viruses, bacteria, and parasites, promote the expansion of MDSCs (20). The ability of MDSCs to dampen effector T-cell responses contributes to their immunosuppressive nature, impacting the overall efficacy of the immune system (21). This, in turn, favors pathogen persistence and the risk of chronicity following acute infection.

In the context of infection, interfering with the inhibitory mechanisms of regulatory cells may assist in the clearance of pathogens. However, a complete understanding of these immunosuppressive mechanisms is required prior to exploiting these novel therapeutic strategies. In this article, we review the mechanisms used by the different regulatory cell types to mediate immunosuppression.

## 2 Mechanisms of Treg-mediated immunosuppression

Tregs inhibit proliferation and production of cytokines after ligation of the receptor [T-cell receptor (TCR)] in effector CD4+ as well as the cytotoxic effect of CD8+ T cells (22, 23). While the main function of Tregs is to prevent excessive immune activation and the maintenance of tolerance to self-antigens (7), they have also been shown to have a significant negative impact on the immune responses to pathogens (11, 12, 24, 25). The diverse functions of Tregs are reflected in the existence of several types, each designated based on their source, generation, and effector mechanisms. The two major subsets identified are the thymus-derived naturally occurring Foxp3+ regulatory T cells (nTregs) and inducible regulatory T cells (iTregs), which develop from peripheral conventional CD4+ T cells in response to stimulus such as microbial products (26). Although both nTregs and iTregs play a significant role in infections due to their ability to control the intensity and duration of the effector responses, natural Tregs play a major role in mediating tolerance to self-antigens and inducible Tregs are the main players in the induction of tolerance to pathogens (27).

Tregs play a crucial and nuanced role in the immune response to various infections (12, 25). For example, in the case of infections caused by *Mycobacterium tuberculosis*, Tregs hinder an effective immune response against the pathogen by inhibiting the production of cytokines like interferon gamma (IFN-γ) or interleukin 17 (IL-17), which are essential for controlling *M. tuberculosis* (28). Indeed, Tregs are expanded in patients infected with *M. tuberculosis* and compromise protective IFN-γ responses and bacterial killing by macrophages (29–31). High amounts of Tregs capable of suppressing antigen-specific production of INF-γ by effector T cells have been found in patients with active tuberculosis (29, 32–34). Tregs have been also shown to expand and restrict bacterial clearance in the lungs of *M. tuberculosis*-infected mice (35). The Tregs arising in *M. tuberculosis*-infected mice proliferated faster than effector T cells and induced delayed recruitment of effector T cells into the infected lungs (36). Tregs have been also shown to suppress protective immunity in other bacterial infections, including those by *Streptococcus pneumoniae* (37), *Salmonella* (38), *Helicobacter pylori* (39), and *Listeria monocytogenes* (40). Tregs play also an important role in the outcome of acute and chronic viral infections, including herpes simplex virus (HSV) (41), human immunodeficiency virus (HIV) (42), hepatitis B virus (HBV) (43), and hepatitis C virus (HCV) (44). Strategies that temporarily dampen the immune-suppressive mechanisms of Tregs could enhance the efficacy of infection therapies, allowing the immune system to mount a more robust response to the infecting agents.

**Figure 1:**
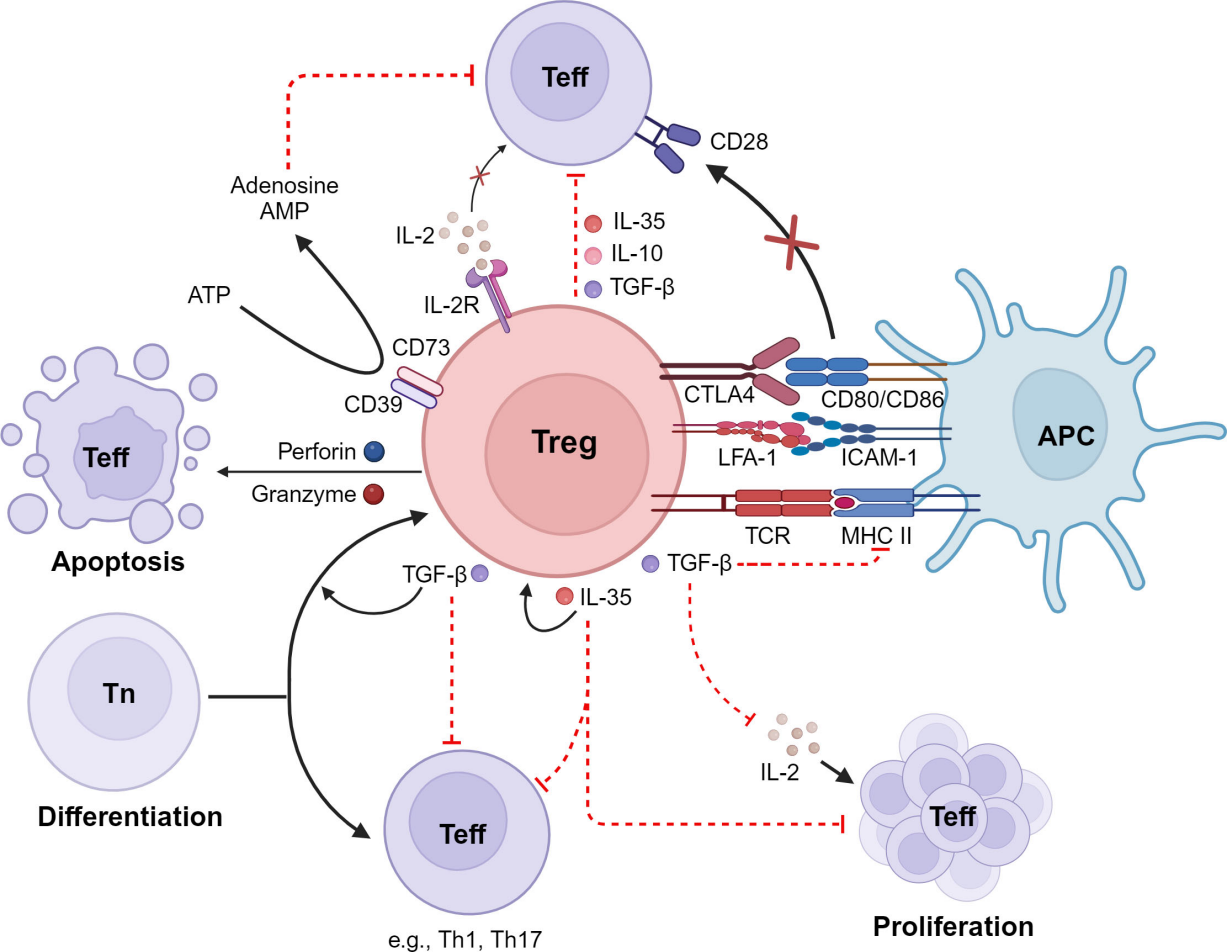
Immunosuppressive mechanisms of Tregs. Tregs inhibit effector T cells (Teff) by 1) the release of inhibitory cytokines, including IL-10, TGF-β, and IL-35; 2) exerting of cytotoxic effects on Teff as well as on antigen-presenting cells (APCs); 3) interference with Teff proliferation via consumption and depletion of IL-2; 4) metabolic disruption; and 5) interference with differentiation of naive T cells (Tn) into Teff. LFA*-*1, lymphocyte function-associated antigen 1; CTLA-4, cytotoxic T-lymphocyte-associated antigen 4; IDO, indoleamine 2,3-dioxygenase; ATP, adenosine triphosphate; cAMP, cyclic adenosine monophosphate. Created with BioRender.com.

Studies in humans and experimental models have revealed that Treg cells employ a variety of mechanisms to suppress immune responses, in both cell contact-dependent and cell contact-independent manners (45, 46). These mechanisms include a) production of suppressive cytokines such as IL-10, transforming growth factor beta (TGF-β), and IL-35; b) induction of cytolysis in effector cells; c) suppression of immune cells or function indirectly by modulating antigen-presenting cells; d) suppression of T cells via IL-2 consumption; and e) generation of immunosuppressive environments through adenosine production (45–47) (**Figure 1**). The different suppressive mechanisms of Tregs are described in more detail in the following sections.

### 2.1 Production of suppressive cytokines

The suppressive cytokines TGF-β and IL-10 have been reported to be involved in Treg-mediated immunosuppression (48–50). The engagement of IL-10 with its receptor on monocytes and macrophages triggers the activation of the Janus kinase/signal transduce and activator of transcription (JAK/STAT) signaling cascade (51). The activation of this pathway by IL-10 results in profound changes in expression of immunomodulatory genes that lead to the inhibition of pro-inflammatory mediator production, decreased antigen presentation capacity, and impaired phagocytosis (51). TGF-β signaling involves activation of suppressor of mothers against decapentaplegic (SMAD) transcription factors (52). TGF-β blocks T helper type 1 (Th1) differentiation and effector functions (53) and silences the expression of IL-2, which is required for T-cell proliferation (54). Furthermore, TGF-β inhibits the antigen presentation capacity of dendritic cells by suppressing expression of major histocompatibility complex (MHC) class II genes (55). Several studies have also reported the ability of Tregs to suppress CD8+ T-lymphocyte cytotoxicity via TGF-β (50, 56) and to suppress differentiation of CD4+ T cells into Th1 effectors (57). Furthermore, TGF-β produced by Tregs induces infectious tolerance by further promoting naive T cells to become immunosuppressive cells, thus leading to long-term propagation of the effects provoked by Tregs (58–61).

Contrary to the perception of TGF-β as a dominant mechanism of Treg suppression, some studies have presented challenges to this notion (62, 63). The findings that the addition of anti-IL-10 or anti-TGF-β antibodies did not impact the suppressive effect of human Treg cells in *in vitro* assays imply that Treg-mediated suppression may involve alternative mechanisms beyond the classical role attributed to IL-10 or TGF-β (64). The diverse functions of IL-10 and TGF-β in Treg-induced suppression in different specific pathologies may provide an explanation for these discrepancies. Addressing these discrepancies and understanding the specific conditions under which these cytokines operate is crucial for a better understanding of Treg function.

IL-35 is an additional inhibitory cytokine that contributes to Treg function (65). IL-35 not only has the ability to directly suppress effector T-cell effector functions and proliferation (65), but it is also able to propagate infectious tolerance by expanding a vigorous population of inducible Tregs (66). Furthermore, IL-35 produced by Tregs can inhibit the capacity of CD4+ T cells to differentiate into Th17 effector cells (67).

### 2.2 Induction of cytolysis in effector cells

Activated human natural Treg cells have been shown to exert cytotoxic activity against various cell types, including monocytes, dendritic cells, and CD4+ and CD8+ T cells (68). This cytotoxic effect is mediated by the perforin/granzyme pathway and is dependent on CD18 adhesive interactions (68). In the perforin/granzyme pathway, perforin and granzymes synergize to mediate apoptosis of target cells such T cells, monocytes, and dendritic cells (69). Thus, perforin induces pores in the target cell membrane and granzymes induce cell death after diffusing into the intracellular compartment through the perforin pores (69). Natural Tregs have been shown to predominantly express granzyme A, whereas iTregs express granzyme B upon activation, but both exert cytotoxicity against autologous targets via perforin (68). Granzyme A and B differ in their target cell-killing mechanism. Granzyme A induces a caspase-independent form of cell death that includes apoptotic features such as DNA damage (70). In contrast, granzyme B triggers apoptosis through a different route by directly cleaving caspases and caspase substrates (71). The differential modes of action of granzyme A and granzyme B exemplify the adaptability of Tregs in utilizing various cytotoxic mechanisms based on the specific context and the nature of the target cell.

### 2.3 Modulation of antigen-presenting cell function

Tregs can also inhibit immune responses by modulating the activity of antigen-presenting cells such as dendritic cells (72–74). In this regard, it has been reported that antigen-specific Tregs can inhibit antigen presentation to T cells by strongly binding to dendritic cells (72). This tight interaction reduces the capacity of dendritic cells to present antigens by promoting the removal of cognate peptide/MHC class II complex (72). Adhesion of Tregs to dendritic cells is mediated by lymphocyte function-associated antigen 1 (LFA*-*1), which exhibits an extraordinarily high strength binding as a consequence of a reduced calpain activities within these cells (73). The decreased calpain activities result in a deficiency in the normal process of integrin recycling, leading to sustained presence of LFA-1 on the cell surface. Consequently, Tregs exhibit prolonged binding to dendritic cells, limiting the physical interactions of dendritic cells with cognate conventional T cells and thereby reducing the capacity of dendritic cells to prime T cells (73).

Co-stimulation by CD28 binding to CD80 and CD86 expressed by antigen-presenting cells is essential for effective T-cell expansion and differentiation (75). Cytotoxic T lymphocyte-associated antigen 4 (CTLA-4) can also bind CD80 and CD86 on antigen-presenting cells, but in contrast to CD28, this molecule is a negative regulator and inhibits T-cell responses (76). Tregs express high levels of CTLA-4, which seems to be an important means of immunosuppression (77–80). Several mechanisms that mediate the inhibitory activity of CTLA-4 have been proposed, including the downregulation of ligand expression and transmission of inhibitory signals (76, 81). Furthermore, CTLA-4 has a superior affinity for CD80 and CD86 molecules than for CD28 (82). By outcompeting with CD28, CTLA-4 downregulates the co-stimulatory signals required for optimal activation of conventional T cells. Tregs can also induce tolerogenic dendritic cells through CTLA-4 engagement-induced tryptophan catabolism (83, 84). Thus, Tregs can stimulate dendritic cells to produce the enzyme indoleamine 2,3-dioxygenase (IDO), which catabolizes the conversion of tryptophan to kynurenine, which is toxic to T cells (85).

### 2.4 Other immunosuppressive mechanisms

Tregs are extremely dependent on IL-2 for their maintenance and functionality, but they lack the capability to produce IL-2 themselves (86–88). Therefore, Tregs rely on the external supply of IL-2, typically provided by activated effector T cells and other immune cells in their microenvironment. Since IL-2 is also critical for the survival and proliferation of effector T cells (89), it has been suggested that one mechanism of Treg suppression of effector T-cell activation is by depriving effector T cells of IL-2 (90–92). An additional suppressing mechanism of Tregs is mediated by the release of high levels of adenosine in the extracellular environment (93). Tregs, in contrast to conventional T cells, express high amounts of CD39 and CD73 on the cell surface, which are nucleotidases capable of producing extracellular adenosine from adenosine triphosphate (ATP) (94–96). Thus, the coordinated action of CD39 and CD72 allows Tregs to generate extracellular adenosine from ATP. The interaction of extracellular adenosine with the adenosine A2A receptor on conventional T cells results in increased cyclic adenosine monophosphate (cAMP) levels, subsequent activation of protein kinase A, and inhibition of T-cell activation (97–99).

## 3 Mechanisms of Breg-mediated immunosuppression

B cells are typically known for their role in the adaptive immune response, including antigen presentation, cytokine secretion, and production of pathogen-specific antibodies (100). However, a subset of B cells with immunomodulatory activity has been identified and termed Bregs (15, 16). Identifying specific phenotypic markers for Bregs has been a challenge, and the characterization of these cells is an area of ongoing research (101). However, several B-cell subsets with regulatory functions have been reported in humans and mice based on their capacity to inhibit effective immune responses *in vivo* or *in vitro* (102). The main Breg subsets identified in humans include CD19+CD24+CD38+ (103) and CD19+CD24^hi^CD27+ (104), and in mice, CD19+CD5+CD1d^hi^ (105), CD5+CD19+B220^low^ (106), and CD19+CD25+CD1d^hi^ IgM^hi^CD5^−^CD23^−^Tim-1^−^ (107). Nevertheless, it is important to note that, rather than relying solely on surface markers, the identification of Bregs is often based on functional assays, such as the ability to produce IL-10 or inhibit immune responses. Ongoing research is focused on gaining a deeper understanding of Breg biology, refining phenotypic markers and identifying markers that are consistently associated with regulatory functions across different contexts.

Generation of Bregs has been reported in a number of infectious diseases, including bacterial, viral, and parasitic infections (108). For example, Bregs have been shown to be involved in the pathogenesis of chronic HBV infection (109) and also to inhibit CD8+ T-cell proliferation and production of IFN-γ in patients infected with HIV (110). Bregs have been implicated in hampering the clearance of hepatitis B virus through the production of IL-10 (111). Also, during bacterial infections such as that by *L. monocytogenes*, expansion of Bregs that inhibit pathogen eradication has been observed in experimental infection in mice (112). A rapid accumulation of Bregs has also been detected in mice infected with *Salmonella typhimurium*, which was detrimental for the course of infection because they inhibited the protective activity mediated by CD4+ T cells, NK cells, and neutrophils (113).

Several mechanisms underlying the regulatory activity of Bregs have been described, including skewing T-cell differentiation toward Tregs (114–116). This skewing process seems to take place by a direct cell–cell interaction between Bregs and T cells as suggested by the requirement of the expression of CD40 and MHC class II (105, 117, 118). It has also been reported that Bregs enter the T-cell zone in lymphoid organs and make more frequent and longer contacts with both CD4+ and CD8+ T cells through direct cognate interaction compared to non-Breg (119). The increased and prolonged interaction between Bregs and T cells reduces the subsequent contacts between T cells and dendritic cells and thereby hinders the process of antigen presentation and subsequent T-cell activation (119). Bregs can also regulate humoral immunity by modulating the activity of follicular helper T cells, which is a population of T cells involved in the activation and differentiation of B cells into antibody-producing plasma cells (120). This effect is mediated by the expression of high levels of programmed death-ligand 1 (PD-L1) on Bregs that binds to PD-1 on T cells (120, 121). Binding of PD-1 to its ligand PD-L1 induces inhibition of the functionality and proliferation of effector T cells (122). However, most of the suppressive activities of Bregs are mediated by the release of high amounts of IL-10. Thus, Bregs can thwart differentiation of T cells toward Th1 or Th17 by inhibition of cytokine production by dendritic cells (123, 124) and promote Th2 cells and Foxp3+ Tregs by producing IL-10 (125, 126). It has been shown that Bregs produce IL-10 after interaction with *Leishmania major*, which leads to downregulation of IL-12 production by dendritic cells, thereby supporting Th2 responses that are detrimental for the proper control of this pathogen (127). Other studies have indicated that direct interaction between Bregs and dendritic cells results in IL-10-mediated deactivation of the dendritic cells, which can result in the suppression of CD8+ T cells (128). Accordingly, by producing IL-10, Bregs have been shown to contribute to the T-cell impairment observed during HIV (110) and chronic hepatitis B virus (109) infections.

**Figure 2:**
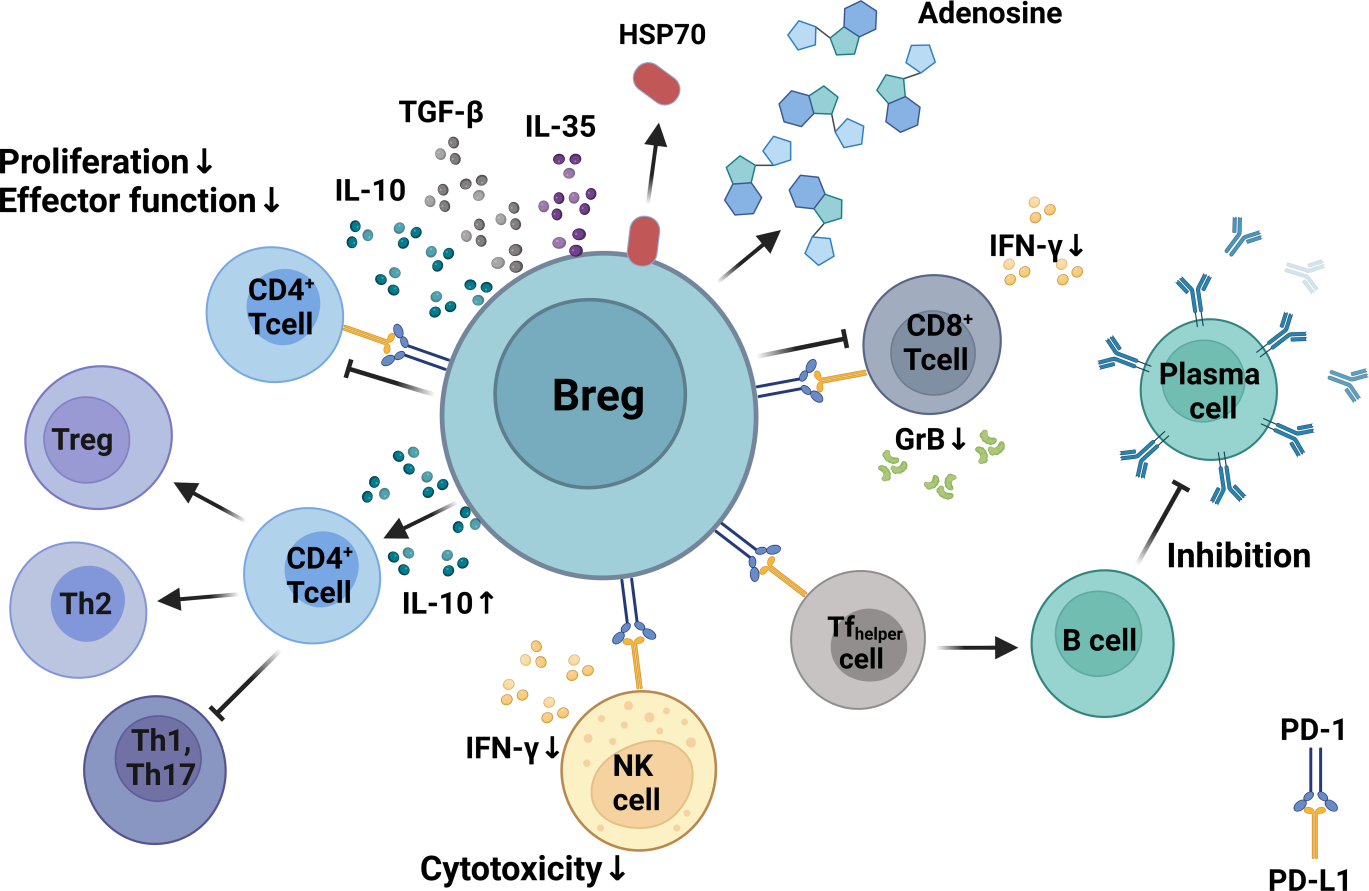
Mechanisms of Breg immunosuppression. Bregs can inhibit the proliferation, differentiation, and functionality of CD4+ and CD8+ T cells via production of IL-10. Bregs can regulate humoral immunity by modulating the activity of follicular T-helper cells via expression of programmed death-ligand 1 (PD-L1) that binds to PD-1 on T cells. Bregs inhibit CD8+ and CD4+ T-cell proliferation and differentiation by producing inhibitory molecules such as IL-10, TGF-β, adenosine, and heat shock protein 70. Bregs inhibit IFN-γ production and suppress the cytotoxicity of CD8+ T cells and NK cells. Created with BioRender.com.

In addition to the release of IL-10, Bregs can also modulate the immune response through the production of other suppressive cytokines such TGF-β and IL-35 (106, 129) as well as other immunomodulatory molecules such as adenosine (130, 131) and heat shock protein 70 (132). The different inhibitory mechanisms of Bregs are illustrated in **Figure 2**.

## 4 Suppressive mechanisms of MDSCs

MDSCs are considered an atypical population of myeloid cells that appear in many pathological disorders, including cancer, autoimmune diseases, and chronic infections, and exert strong suppressive activity on T cells (133). MDSCs originate from common myeloid progenitors but they do not undergo full maturation and remain in an immature differentiation status (19, 134). Phenotypically, MDSCs are commonly divided into two different subsets, monocytic and granulocytic, based on the expression of CD14+CD11b+CD33+HLA-DR− and CD15+CD11b+CD33+HLA-DR-, respectively, in humans (135). The phenotypic markers for murine monocytic MDSCs are CD11b+Ly6C+Ly6G^low^ and CD11b+Ly6C^low^Ly6G+ for granulocytic MDSC (135). However, these markers are not exclusive to MDSCs and are also expressed by mature monocytes, neutrophils, and other hematopoietic precursor lineages (136). Additional markers such as the chemokine CCL6 have been identified in the murine system that enable to discriminate immature granulocytes precursors (Ly6G^+^CCL6^−^) from mature neutrophils (Ly6G^+^CCL6^+^) (134). Despite the additional markers, differentiation of MDSCs from other myeloid cells based on these phenotypic markers is rather challenging and functional assays that confirm their immunosuppressive activity are essential for a more definitive assessment. Furthermore, while monocytic and granulocytic subsets are commonly recognized, additional subsets and phenotypic variations have been described in various studies (137). The high degree of heterogeneity within the MDSC population has been clearly illustrated in the single-cell RNA sequencing (RNA-seq) analysis of MDSCs generated in mice during chronic *Staphylococcus aureus* infection performed in our laboratory. This analysis shows that the population of MDSCs comprised a continuum of myeloid cell precursors in different differentiation stages (134). The spectrum of myeloid cell precursors within the MDSC population can extend to earlier stages of myeloid differentiation, involving common myeloid progenitors. Expansion of MDSCs in the context of infection may be associated with emergency granulopoiesis, which involves a rapid release of immature myeloid cells into the circulation in response to the need for an elevated production of myeloid cells to combat the infection (134).

MDSCs are known for their ability to suppress various components of the immune system, extending beyond T cells (138–142). They can exert inhibitory effects on other immune cell types, including B cells and NK cells (138–142). Many pathogens, including bacteria and viruses, promote expansion of MDSCs as a means of suppressing the immune response mounted by the host (20). In this regard, expansion of MDSCs has been associated with tuberculosis progression in humans (143) and mice (144, 145). The induction of MDSCs in response to *M. tuberculosis* has been implicated in the impaired ability of the host to eliminate the bacterium, thereby contributing to the development of tuberculosis disease (143). MDSCs have been reported to play an important role in chronic infections caused by *S. aureus*, a notorious pathogen known for its ability to cause challenging and difficult-to-treat chronic infections (146, 147). Thus, expansion of MDSCs has been linked to progressive dysfunction of T cells and failure to eliminate *S. aureus* in murine models of staphylococcal chronic abscess (146). In infected prosthetic joints, MDSCs have been shown to inhibit the pro-inflammatory activity of monocytes/macrophages, thereby facilitating the chronicity of *S. aureus* orthopedic biofilm infection (147). Increased frequency of MDSCs that inhibit protective T-cell responses via nitric oxide production has been also reported in mice infected with *Salmonella enterica* serovar Typhimurium (148).

The generation of MDSCs in many viral infections seems to contribute to the establishment of a chronic course (149, 150). Thus, immunosuppression of T-cell responses mediated by reactive oxygen species (ROS) produced by MDSCs has been shown to initiate and maintain HCV persistence (151). MDSCs also inhibit the production of IFN-γ, a key cytokine involved in anti-viral defense, by natural killer cells in patients infected with HCV via an arginase-1-dependent mechanism (140). Several studies have also reported elevated numbers of MDSCs in patients with chronic HIV infection, which dampen anti-HIV T-cell-mediated immune responses (152, 153) and promote the development of Tregs (154). An increased frequency of MDSCs has been observed in the peripheral blood of patients infected with severe acute respiratory syndrome coronavirus 2 (SARS-CoV-2), particularly those with severe disease (155). The expansion of MDSCs in SARS-CoV-2-infected patients appears to correlate with the severity of respiratory symptoms and the need for intensive care (155).

**Figure 3:**
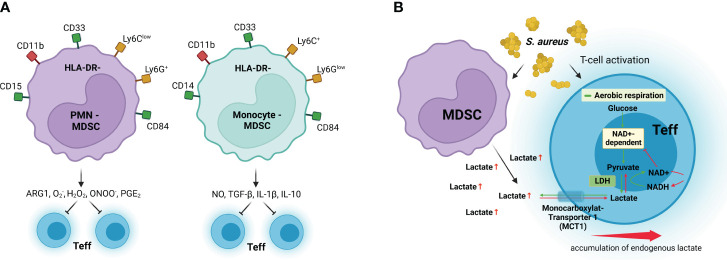
MDSC immunosuppressive mechanisms. **(A)** Granulocytic MDSCs (PMN-MDSC) preferentially use arginase 1 (ARG1), reactive oxygen species such as superoxide (O_2_^−^) and hydrogen peroxide (H_2_O_2_), and peroxynitrite (ONOO^−^) to inhibit effector T-cell (Teff) responses, whereas monocytic MDSCs (Monocyte-MDSC) preferentially use nitric oxide (NO), inhibitory cytokines such as TGF-β and IL-10, and the receptors CTLA-4 and PD-1, which induce anergy and apoptosis after binding their respective receptors in Teff. **(B)** MDSCs inhibit T-cell activation by excretion of high levels of lactate, which results in discontinuous glycolysis and impedes NAD+ regeneration from NADH in Teff. LDH, lactate dehydrogenase; NAD+, oxidized nicotinamide adenine dinucleotide; NADH, reduced nicotinamide adenine dinucleotide; MCT1, monocarboxylate transporter 1. Surface receptors of human MDSCs are shown in green and red, and surface receptors for murine MDSCs are shown in yellow and red. Created with BioRender.com.

The mechanisms implicated in the suppressive activity of MDSC in the context of infections are described in the following section and summarized in **Figures 3A, B**.

### 4.1 Suppression mediated by arginine metabolism

L-Arginine is an essential amino acid that is critical for body physiology because it is required for protein synthesis and for the production of nitric oxide, creatine, and polyamines (156). L-Arginine accessibility is crucial for activation and proper functionality of T cells (157). Arginine can be metabolized either to nitric oxide by the activity of the nitric oxide synthase or to urea and L-ornithine by the activity of arginase enzymes (158). Metabolism of L-arginine by arginase-1 has been reported to be a substantial mechanism of MDSC suppression of T-cell responses by depleting L-arginine in the T-cell microenvironment (159, 160). L-Arginine starvation hampers T-cell responses by provoking an arrest in the proliferation of activated T cells (161) as well as by reducing the expression of the CD3ζ chain (162, 163).

### 4.2 Suppression mediated by nitric oxide and reactive oxygen species

Nitric oxide produced in large amounts by MDSCs via arginase activity can suppress T-cell responses (164) and can obstruct T-cell migration by inhibiting vascular expression of E-selectin (165). Furthermore, nitric oxide has been suggested to trigger suppression of T-cell responses by altering key molecules in the signaling pathway induced after IL-2 binding to its surface receptor (166). Nitric oxide can also affect the stability of the IL-2 messenger RNA (mRNA), resulting in reduced IL-2 release by T cells (167).

MDSCs can also generate high levels of ROS, including hydrogen peroxide (H_2_O_2_), peroxynitrite (ONOO^−^), and superoxide (O2^−^), which can have damaging effects on nucleic acids, lipids, and proteins (168). Thus, ROS produced by MDSCs have been shown to suppress antigen-specific CD8+ T cells by inducing alterations in the T-cell receptor that impair the capacity of CD8+ T cells to bind MHC class I on antigen-presenting cells (169). MDSCs are capable of avoiding the toxic effects of the high levels of ROS that they generate by upregulating series of genes via nuclear factor erythroid 2-related factor 2 (Nrf2) that mitigate oxidative stress (170).

Similar to other regulatory cell populations described in the previous sections, MDSCs can also suppress T-cell responses by production of inhibitory cytokines such as IL-10 and TGF-β (141, 171, 172). For example, it has been reported that TGF-β produced by MDSCs hinders the functionality of NK cells by inhibiting their capacity to produce IFN-γ as well as their cytotoxic activity (141). Furthermore, MDSCs were shown to be able to induce other immunosuppressive cells such as Tregs in HIV-infected individuals (154).

### 4.3 Suppression by altering T-cell metabolism

Upon antigen recognition and activation via TCR, effector T cells proliferate extensively and develop effector functions. Through the activation process, T cells need to reprogram their metabolism from oxidative phosphorylation toward aerobic glycolysis to ensure the bioenergetic demands required for cell division and production of effector molecules (173–175). Glycolysis is the major pathway of glucose metabolism. In resting cells and under aerobic conditions, glucose is usually converted into pyruvate, which is further oxidized to generate acetyl-coenzyme A, which enters the mitochondria and undergoes further oxidation in the citric acid cycle. In the absence of oxygen, pyruvate is converted to lactate instead of entering the mitochondria to undergo oxidation. In proliferating cells, a significant portion of pyruvate is converted to lactate in the cytoplasm even in the presence of oxygen rather than entering the mitochondria and undergoing complete oxidation. This reaction is known as “aerobic glycolysis” (175). This is considered an adaptation to the rapid growth and high energy demands of proliferating cells (173–175). By using aerobic glycolysis, cells can quickly generate ATP and metabolic intermediates needed for the synthesis of macromolecules such as nucleotides, amino acids, and lipids, which are crucial for cell proliferation (173–175). During the conversion of pyruvate to lactate in aerobic glycolysis, reduced nicotinamide adenine dinucleotide (NADH) donates electrons to pyruvate, converting it to lactate and regenerating oxidized nicotinamide adenine dinucleotide (NAD+) (175). This process is essential during glycolysis and other metabolic pathways where NAD+ serves as a crucial cofactor for many enzymes (175). At the same time, the excess of lactate produced during aerobic glycolysis in proliferating cells needs to be exported from the cells to prevent the buildup of lactate, which could otherwise inhibit glycolysis. This export is facilitated by proton-linked monocarboxylate transporters that are dependent on a concentration gradient. Our group has reported that MDSCs generated during chronic *S. aureus* infection in mice exhibit elevated glycolytic activity and release a high amount of lactate in the extracellular microenvironment (134). In further studies, we demonstrated that the high levels of lactate discharged by MDSCs change the transmembrane concentration gradient and inhibit lactate removal by activated CD4+ T cells (176). This results in an intracellular buildup of lactate that hinders the regeneration of NAD+, inhibits the activity of NAD-dependent glycolytic enzymes, and discontinues glycolysis (176). Therefore, an important mechanism of T-cell immunosuppression by MDSCs is disturbing their capacity to undergo metabolic reprogramming.

## 5 Clinical relevance and future perspectives

Targeting regulatory cells could be an attractive option for the therapy of infectious diseases, in particular those with a chronic course. However, elimination of regulatory cells could lead to immune dysregulation, contributing to the development of autoimmune diseases, inflammatory conditions, and risk of tissue damage caused by an overactive immune system. Therefore, modulation of the mechanisms mediating the immune-suppressive effect of regulatory cells may provide a more nuanced approach compared to direct elimination of these cells. For example, several regulatory cell subsets often exert their suppressive effects through the secretion of immunosuppressive cytokines, such as IL-10 and TGF-β. Targeting these cytokines or their receptors could be a strategy to modulate their suppressive activity. Surface molecules on regulatory cells, such as CTLA-4 and PD-1, are involved in immune suppression. Blocking these molecules or their ligands can disrupt the inhibitory signals. These strategies would enable the fine-tuning of the immune response to infection, promoting an appropriate and controlled reaction to pathogens while maintaining immune homeostasis. Additionally, targeting specific mechanisms should be context specific and consider the individual characteristics of the different infections. In this regard, precision medicine approaches that target suppressive mechanisms in a controlled and selective manner are desired. However, considering the wide spectrum of immunosuppressive mechanisms with redundant and overlapping functions in the different subsets of regulatory cells, it is possible to anticipate that one single strategy might not be sufficient to mount proper immune responses and effective immunotherapies will require multifaceted approaches. Therefore, effective immunotherapies will require combinatorial regimens to restore cell effector functions and improve the infection outcome by not only mitigating the effect of immunosuppressive mechanisms but also incorporating methods to control tissue damage produced by excessive inflammation.

The complexity of the regulatory network in most infections is a challenging yet crucial area of research. Understanding the intricate details of the immune regulatory mechanisms during infections can unveil critically important features that can inform the development of targeted therapies and enhance our ability to manipulate immune responses for improved outcomes in infectious diseases, in particular those with a persistent or chronic course.

## Author contributions

OG: Writing – review & editing, Conceptualization, Writing – original draft. ON: Writing – review & editing, Writing – original draft. QC: Writing – review & editing, Writing – original draft. EM: Funding acquisition, Writing – original draft, Conceptualization, Writing – review & editing.

## References

[1] Fajgenbaum DC June CH. Cytokine storm. N Engl J Med (2020) 383(23):2255–73. doi: 10.1056/NEJMra2026131 PMC7727315 33264547

[2] Zuniga EI Macal M Lewis GM Harker JA. Innate and adaptive immune regulation during chronic viral infections. Annu Rev Virol (2015) 2(1):573–97. doi: 10.1146/annurev-virology-100114-055226 PMC4785831 26958929

[3] Papp G Boros P Nakken B Szodoray P Zeher M. Regulatory immune cells and functions in autoimmunity and transplantation immunology. Autoimmun Rev (2017) 16(5):435–44. doi: 10.1016/j.autrev.2017.03.011 28286106

[4] Josefowicz SZ Lu LF Rudensky AY. Regulatory T cells: mechanisms of differentiation and function. Annu Rev Immunol (2012) 30:531–64. doi: 10.1146/annurev.immunol.25.022106.141623 PMC6066374 22224781

[5] Sakaguchi S Mikami N Wing JB Tanaka A Ichiyama K Ohkura N. Regulatory T cells and human disease. Annu Rev Immunol (2020) 38:541–66. doi: 10.1146/annurev-immunol-042718-041717 32017635

[6] Kim JM Rasmussen JP Rudensky AY. Regulatory T cells prevent catastrophic autoimmunity throughout the lifespan of mice. Nat Immunol (2007) 8(2):191–7. doi: 10.1038/ni1428 17136045

[7] Sakaguchi S Yamaguchi T Nomura T Ono M. Regulatory T cells and immune tolerance. Cell (2008) 133(5):775–87. doi: 10.1016/j.cell.2008.05.009 18510923

[8] Gavin MA Rasmussen JP Fontenot JD Vasta V Manganiello VC Beavo JA. Foxp3-dependent programme of regulatory T-cell differentiation. Nature (2007) 445(7129):771–5. doi: 10.1038/nature05543 17220874

[9] Hori S Nomura T Sakaguchi S. Control of regulatory T cell development by the transcription factor foxp3. Science (2003) 299(5609):1057–61. doi: 10.1126/science.1079490 12522256

[10] Fontenot JD Gavin MA Rudensky AY. Foxp3 programs the development and function of cd4+Cd25+ Regulatory T cells. Nat Immunol (2003) 4(4):330–6. doi: 10.1038/ni904 12612578

[11] Belkaid Y Tarbell K. Regulatory T cells in the control of host-microorganism interactions. Annu Rev Immunol (2009) 27:551–89. doi: 10.1146/annurev.immunol.021908.132723 19302048

[12] Belkaid Y. Regulatory T cells and infection: A dangerous necessity. Nat Rev Immunol (2007) 7(11):875–88. doi: 10.1038/nri2189 17948021

[13] LeBien TW Tedder TF. B lymphocytes: how they develop and function. Blood (2008) 112(5):1570–80. doi: 10.1182/blood-2008-02-078071 PMC2518873 18725575

[14] Berthelot JM Jamin C Amrouche K Le Goff B Maugars Y Youinou P. Regulatory B cells play a key role in immune system balance. Joint Bone Spine (2013) 80(1):18–22. doi: 10.1016/j.jbspin.2012.04.010 22858147

[15] Mauri C Bosma A. Immune regulatory function of B cells. Annu Rev Immunol (2012) 30:221–41. doi: 10.1146/annurev-immunol-020711-074934 22224776

[16] Rosser EC Mauri C. Regulatory B cells: origin, phenotype, and function. Immunity (2015) 42(4):607–12. doi: 10.1016/j.immuni.2015.04.005 25902480

[17] Dai YC Zhong J Xu JF. Regulatory B cells in infectious disease. Mol Med Rep (2017) 16(1):3–10. doi: 10.3892/mmr.2017.6605 28534949 PMC5482109

[18] Dorhoi A Glaria E Garcia-Tellez T Nieuwenhuizen NE Zelinskyy G Favier B. MDSC in infectious diseases: regulation, roles, and readjustment. Cancer Immunol Immunother (2019) 68(4):673–85. doi: 10.1007/s00262-018-2277-y PMC11028159 30569204

[19] Gabrilovich DI Nagaraj S. Myeloid-derived suppressor cells as regulators of the immune system. Nat Rev Immunol (2009) 9(3):162–74. doi: 10.1038/nri2506 PMC2828349 19197294

[20] Medina E Hartl D. Myeloid-derived suppressor cells in infection: A general overview. J Innate Immun (2018) 10(5-6):407–13. doi: 10.1159/000489830 PMC6784037 29945134

[21] Talmadge JE Gabrilovich DI. History of myeloid-derived suppressor cells. Nat Rev Cancer (2013) 13(10):739–52. doi: 10.1038/nrc3581 PMC4358792 24060865

[22] Thornton AM Shevach EM. Cd4+Cd25+ Immunoregulatory T cells suppress polyclonal T cell activation in vitro by inhibiting interleukin 2 production. J Exp Med (1998) 188(2):287–96. doi: 10.1084/jem.188.2.287 PMC2212461 9670041

[23] Suri-Payer E Amar AZ Thornton AM Shevach EM. Cd4+Cd25+ T cells inhibit both the induction and effector function of autoreactive T cells and represent a unique lineage of immunoregulatory cells. J Immunol (1998) 160(3):1212–8. doi: 10.4049/jimmunol.160.3.1212 9570536

[24] Keynan Y Card CM McLaren PJ Dawood MR Kasper K Fowke KR. The role of regulatory T cells in chronic and acute viral infections. Clin Infect Dis (2008) 46(7):1046–52. doi: 10.1086/529379 18444822

[25] Maizels RM Smith KA. Regulatory T cells in infection. Adv Immunol (2011) 112:73–136. doi: 10.1016/B978-0-12-387827-4.00003-6 22118407 PMC7150045

[26] O’Garra A Vieira PL Vieira P Goldfeld AE. IL-10 producing and naturally occurring cd4+ Tregs: limiting collateral damage. J Clin Invest (2004) 114(10):1372–8. doi: 10.1172/JCI23215 PMC525746 15545984

[27] Shevach EM Thornton AM. tTregs, pTregs, and iTregs: similarities and differences. Immunol Rev (2014) 259(1):88–102. doi: 10.1111/imr.12160 24712461 PMC3982187

[28] Cardona P Cardona PJ. Regulatory T cells in *Mycobacterium tuberculosis* infection. Front Immunol (2019) 10:2139. doi: 10.3389/fimmu.2019.02139 31572365 PMC6749097

[29] Guyot-Revol V Innes JA Hackforth S Hinks T Lalvani A. Regulatory T cells are expanded in blood and disease sites in patients with tuberculosis. Am J Respir Crit Care Med (2006) 173(7):803–10. doi: 10.1164/rccm.200508-1294OC 16339919

[30] Hougardy JM Place S Hildebrand M Drowart A Debrie AS Locht C. Regulatory T cells depress immune responses to protective antigens in active tuberculosis. Am J Respir Crit Care Med (2007) 176(4):409–16. doi: 10.1164/rccm.200701-084OC 17541018

[31] Semple PL Binder AB Davids M Maredza A van Zyl-Smit RN Dheda K. Regulatory T cells attenuate mycobacterial stasis in alveolar and blood-derived macrophages from patients with tuberculosis. Am J Respir Crit Care Med (2013) 187(11):1249–58. doi: 10.1164/rccm.201210-1934OC 23590266

[32] Zewdie M Howe R Hoff ST Doherty TM Getachew N Tarekegne A. Ex-vivo characterization of regulatory T cells in pulmonary tuberculosis patients, latently infected persons, and healthy endemic controls. Tuberculosis (Edinb) (2016) 100:61–8. doi: 10.1016/j.tube.2016.06.007 PMC5111553 27553411

[33] Chen X Zhou B Li M Deng Q Wu X Le X. Cd4(+)Cd25(+)Foxp3(+) regulatory T cells suppress *Mycobacterium tuberculosis* immunity in patients with active disease. Clin Immunol (2007) 123(1):50–9. doi: 10.1016/j.clim.2006.11.009 17234458

[34] Li L Lao SH Wu CY. Increased frequency of cd4(+)Cd25(High) Treg cells inhibit BCG-specific induction of IFN-gamma by cd4(+) T cells from tb patients. Tuberculosis (Edinb) (2007) 87(6):526–34. doi: 10.1016/j.tube.2007.07.004 17851131

[35] Kursar M Koch M Mittrucker HW Nouailles G Bonhagen K Kamradt T. Cutting edge: regulatory T cells prevent efficient clearance of *Mycobacterium tuberculosis* . J Immunol (2007) 178(5):2661–5. doi: 10.4049/jimmunol.178.5.2661 17312107

[36] Shafiani S Tucker-Heard G Kariyone A Takatsu K Urdahl KB. Pathogen-specific regulatory T cells delay the arrival of effector T cells in the lung during early tuberculosis. J Exp Med (2010) 207(7):1409–20. doi: 10.1084/jem.20091885 PMC2901066 20547826

[37] Zhang Q Leong SC McNamara PS Mubarak A Malley R Finn A. Characterisation of regulatory T cells in nasal associated lymphoid tissue in children: relationships with pneumococcal colonization. PloS Pathog (2011) 7(8):e1002175. doi: 10.1371/journal.ppat.1002175 21852948 PMC3154846

[38] Johanns TM Ertelt JM Rowe JH Way SS. Regulatory T cell suppressive potency dictates the balance between bacterial proliferation and clearance during persistent Salmonella infection. PloS Pathog (2010) 6(8):e1001043. doi: 10.1371/journal.ppat.1001043 20714351 PMC2920851

[39] Robinson K Kenefeck R Pidgeon EL Shakib S Patel S Polson RJ. *Helicobacter pylori*-induced peptic ulcer disease is associated with inadequate regulatory T cell responses. Gut (2008) 57(10):1375–85. doi: 10.1136/gut.2007.137539 18467372

[40] Rowe JH Ertelt JM Aguilera MN Farrar MA Way SS. Foxp3(+) regulatory T cell expansion required for sustaining pregnancy compromises host defense against prenatal bacterial pathogens. Cell Host Microbe (2011) 10(1):54–64. doi: 10.1016/j.chom.2011.06.005 21767812 PMC3140139

[41] Suvas S Kumaraguru U Pack CD Lee S Rouse BT. Cd4+Cd25+ T cells regulate virus-specific primary and memory cd8+ T cell responses. J Exp Med (2003) 198(6):889–901. doi: 10.1084/jem.20030171 12975455 PMC2194203

[42] Kinter A McNally J Riggin L Jackson R Roby G Fauci AS. Suppression of HIV-specific T cell activity by lymph node cd25+ Regulatory T cells from HIV-infected individuals. Proc Natl Acad Sci USA (2007) 104(9):3390–5. doi: 10.1073/pnas.0611423104 PMC1805624 17360656

[43] Xu D Fu J Jin L Zhang H Zhou C Zou Z. Circulating and liver resident cd4+Cd25+ Regulatory T cells actively influence the antiviral immune response and disease progression in patients with hepatitis B. J Immunol (2006) 177(1):739–47. doi: 10.4049/jimmunol.177.1.739 16785573

[44] Cabrera R Tu Z Xu Y Firpi RJ Rosen HR Liu C. An immunomodulatory role for cd4(+)Cd25(+) regulatory T lymphocytes in hepatitis C virus infection. Hepatology (2004) 40(5):1062–71. doi: 10.1002/hep.20454 15486925

[45] Schmidt A Oberle N Krammer PH. Molecular mechanisms of Treg-mediated T cell suppression. Front Immunol (2012) 3:51. doi: 10.3389/fimmu.2012.00051 22566933 PMC3341960

[46] Vignali DA Collison LW Workman CJ. How regulatory T cells work. Nat Rev Immunol (2008) 8(7):523–32. doi: 10.1038/nri2343 PMC2665249 18566595

[47] Sojka DK Huang YH Fowell DJ. Mechanisms of regulatory T-cell suppression - a diverse arsenal for a moving target. Immunology (2008) 124(1):13–22. doi: 10.1111/j.1365-2567.2008.02813.x 18346152 PMC2434375

[48] Asseman C Mauze S Leach MW Coffman RL Powrie F. An essential role for interleukin 10 in the function of regulatory T cells that inhibit intestinal inflammation. J Exp Med (1999) 190(7):995–1004. doi: 10.1084/jem.190.7.995 10510089 PMC2195650

[49] Powrie F Carlino J Leach MW Mauze S Coffman RL. A critical role for transforming growth factor-beta but not interleukin 4 in the suppression of T helper type 1-mediated colitis by cd45rb(Low) cd4+ T cells. J Exp Med (1996) 183(6):2669–74. doi: 10.1084/jem.183.6.2669 PMC2192626 8676088

[50] Chen ML Pittet MJ Gorelik L Flavell RA Weissleder R von Boehmer H. Regulatory T cells suppress tumor-specific cd8 T cell cytotoxicity through TGF-beta signals in vivo. Proc Natl Acad Sci USA (2005) 102(2):419–24. doi: 10.1073/pnas.0408197102 PMC544311 15623559

[51] Iyer SS Cheng G. Role of interleukin 10 transcriptional regulation in inflammation and autoimmune disease. Crit Rev Immunol (2012) 32(1):23–63. doi: 10.1615/critrevimmunol.v32.i1.30 22428854 PMC3410706

[52] Batlle E Massague J. Transforming growth factor-beta signaling in immunity and cancer. Immunity (2019) 50(4):924–40. doi: 10.1016/j.immuni.2019.03.024 PMC7507121 30995507

[53] Li MO Wan YY Sanjabi S Robertson AK Flavell RA. Transforming growth factor-beta regulation of immune responses. Annu Rev Immunol (2006) 24:99–146. doi: 10.1146/annurev.immunol.24.021605.090737 16551245

[54] Brabletz T Pfeuffer I Schorr E Siebelt F Wirth T Serfling E. Transforming growth factor beta and cyclosporin a inhibit the inducible activity of the interleukin-2 gene in T cells through a noncanonical octamer-binding site. Mol Cell Biol (1993) 13(2):1155–62. doi: 10.1128/mcb.13.2.1155-1162.1993 PMC359000 8423782

[55] Nandan D Reiner NE. TGF-beta attenuates the class II transactivator and reveals an accessory pathway of IFN-gamma action. J Immunol (1997) 158(3):1095–101. doi: 10.4049/jimmunol.158.3.1095 9013947

[56] Mempel TR Pittet MJ Khazaie K Weninger W Weissleder R von Boehmer H. Regulatory T cells reversibly suppress cytotoxic T cell function independent of effector differentiation. Immunity (2006) 25(1):129–41. doi: 10.1016/j.immuni.2006.04.015 16860762

[57] Shen E Zhao K Wu C Yang B. The suppressive effect of cd25+Treg cells on Th1 differentiation requires cell-cell contact partially via TGF-beta production. Cell Biol Int (2011) 35(7):705–12. doi: 10.1042/CBI20100528 21314640

[58] Jonuleit H Schmitt E Kakirman H Stassen M Knop J Enk AH. Infectious tolerance: human cd25(+) regulatory T cells convey suppressor activity to conventional cd4(+) T helper cells. J Exp Med (2002) 196(2):255–60. doi: 10.1084/jem.20020394 PMC2193929 12119350

[59] Zheng SG Wang JH Gray JD Soucier H Horwitz DA. Natural and induced cd4+Cd25+ Cells educate cd4+Cd25- cells to develop suppressive activity: the role of IL-2, TGF-beta, and IL-10. J Immunol (2004) 172(9):5213–21. doi: 10.4049/jimmunol.172.9.5213 15100259

[60] Qiao M Thornton AM Shevach EM. Cd4+ Cd25+ [Corrected] regulatory T cells render naive cd4+ Cd25- T cells anergic and suppressive. Immunology (2007) 120(4):447–55. doi: 10.1111/j.1365-2567.2007.02544.x PMC2265911 17244157

[61] Andersson J Tran DQ Pesu M Davidson TS Ramsey H O’Shea JJ. Cd4+ Foxp3+ Regulatory T cells confer infectious tolerance in a TGF-beta-dependent manner. J Exp Med (2008) 205(9):1975–81. doi: 10.1084/jem.20080308 PMC2526184 18710931

[62] Piccirillo CA Letterio JJ Thornton AM McHugh RS Mamura M Mizuhara H. Cd4(+)Cd25(+) regulatory T cells can mediate suppressor function in the absence of transforming growth factor beta1 production and responsiveness. J Exp Med (2002) 196(2):237–46. doi: 10.1084/jem.20020590 PMC2193919 12119348

[63] Oberle N Eberhardt N Falk CS Krammer PH Suri-Payer E. Rapid suppression of cytokine transcription in human cd4+Cd25 T cells by cd4+Foxp3+ Regulatory T cells: independence of IL-2 consumption, TGF-beta, and various inhibitors of TCR signaling. J Immunol (2007) 179(6):3578–87. doi: 10.4049/jimmunol.179.6.3578 17785792

[64] Jonuleit H Schmitt E Stassen M Tuettenberg A Knop J Enk AH. Identification and functional characterization of human cd4(+)Cd25(+) T cells with regulatory properties isolated from peripheral blood. J Exp Med (2001) 193(11):1285–94. doi: 10.1084/jem.193.11.1285 PMC2193380 11390435

[65] Collison LW Workman CJ Kuo TT Boyd K Wang Y Vignali KM. The inhibitory cytokine IL-35 contributes to regulatory T-cell function. Nature (2007) 450(7169):566–9. doi: 10.1038/nature06306 18033300

[66] Olson BM Sullivan JA Burlingham WJ. Interleukin 35: A key mediator of suppression and the propagation of infectious tolerance. Front Immunol (2013) 4:315. doi: 10.3389/fimmu.2013.00315 24151492 PMC3798782

[67] Niedbala W Wei XQ Cai B Hueber AJ Leung BP McInnes IB. IL-35 Is a Novel Cytokine with Therapeutic Effects against Collagen-Induced Arthritis through the Expansion of Regulatory T Cells and Suppression of Th17 Cells. Eur J Immunol (2007) 37(11):3021–9. doi: 10.1002/eji.200737810 17874423

[68] Grossman WJ Verbsky JW Barchet W Colonna M Atkinson JP Ley TJ. Human T regulatory cells can use the perforin pathway to cause autologous target cell death. Immunity (2004) 21(4):589–601. doi: 10.1016/j.immuni.2004.09.002 15485635

[69] Voskoboinik I Whisstock JC Trapani JA. Perforin and granzymes: function, dysfunction and human pathology. Nat Rev Immunol (2015) 15(6):388–400. doi: 10.1038/nri3839 25998963

[70] Lieberman J. Granzyme a activates another way to die. Immunol Rev (2010) 235(1):93–104. doi: 10.1111/j.0105-2896.2010.00902.x 20536557 PMC2905780

[71] Chowdhury D Lieberman J. Death by a thousand cuts: granzyme pathways of programmed cell death. Annu Rev Immunol (2008) 26:389–420. doi: 10.1146/annurev.immunol.26.021607.090404 18304003 PMC2790083

[72] Akkaya B Oya Y Akkaya M Al Souz J Holstein AH Kamenyeva O. Regulatory T cells mediate specific suppression by depleting peptide-MHC class II from dendritic cells. Nat Immunol (2019) 20(2):218–31. doi: 10.1038/s41590-018-0280-2 PMC6402611 30643268

[73] Chen J Ganguly A Mucsi AD Meng J Yan J Detampel P. Strong adhesion by regulatory T cells induces dendritic cell cytoskeletal polarization and contact-dependent lethargy. J Exp Med (2017) 214(2):327–38. doi: 10.1084/jem.20160620 PMC5294852 28082358

[74] Yan J Liu B Shi Y Qi H. Class II MHC-independent suppressive adhesion of dendritic cells by regulatory T cells in vivo. J Exp Med (2017) 214(2):319–26. doi: 10.1084/jem.20160629 PMC5294853 28082359

[75] Chen L Flies DB. Molecular mechanisms of T cell co-stimulation and co-inhibition. Nat Rev Immunol (2013) 13(4):227–42. doi: 10.1038/nri3405 PMC3786574 23470321

[76] Walker LS Sansom DM. The emerging role of CTLA-4 as a cell-extrinsic regulator of T cell responses. Nat Rev Immunol (2011) 11(12):852–63. doi: 10.1038/nri3108 22116087

[77] Read S Greenwald R Izcue A Robinson N Mandelbrot D Francisco L. Blockade of CTLA-4 on cd4+Cd25+ Regulatory T cells abrogates their function in vivo. J Immunol (2006) 177(7):4376–83. doi: 10.4049/jimmunol.177.7.4376 PMC6108417 16982872

[78] Wing K Onishi Y Prieto-Martin P Yamaguchi T Miyara M Fehervari Z. CTLA-4 control over foxp3+ Regulatory T cell function. Science (2008) 322(5899):271–5. doi: 10.1126/science.1160062 18845758

[79] Ise W Kohyama M Nutsch KM Lee HM Suri A Unanue ER. CTLA-4 suppresses the pathogenicity of self antigen-specific T cells by cell-intrinsic and cell-extrinsic mechanisms. Nat Immunol (2010) 11(2):129–35. doi: 10.1038/ni.1835 PMC3235641 20037585

[80] Tai X Van Laethem F Pobezinsky L Guinter T Sharrow SO Adams A. Basis of CTLA-4 function in regulatory and conventional cd4(+) T cells. Blood (2012) 119(22):5155–63. doi: 10.1182/blood-2011-11-388918 PMC3369608 22403258

[81] Rudd CE Taylor A Schneider H. Cd28 and CTLA-4 coreceptor expression and signal transduction. Immunol Rev (2009) 229(1):12–26. doi: 10.1111/j.1600-065X.2009.00770.x 19426212 PMC4186963

[82] Collins AV Brodie DW Gilbert RJ Iaboni A Manso-Sancho R Walse B. The interaction properties of costimulatory molecules revisited. Immunity (2002) 17(2):201–10. doi: 10.1016/s1074-7613(02)00362-x 12196291

[83] Fallarino F Grohmann U Hwang KW Orabona C Vacca C Bianchi R. Modulation of tryptophan catabolism by regulatory T cells. Nat Immunol (2003) 4(12):1206–12. doi: 10.1038/ni1003 14578884

[84] Grohmann U Orabona C Fallarino F Vacca C Calcinaro F Falorni A. CTLA-4-Ig regulates tryptophan catabolism *in vivo* . Nat Immunol (2002) 3(11):1097–101. doi: 10.1038/ni846 12368911

[85] Terness P Bauer TM Rose L Dufter C Watzlik A Simon H. Inhibition of allogeneic T cell proliferation by indoleamine 2,3-dioxygenase-expressing dendritic cells: mediation of suppression by tryptophan metabolites. J Exp Med (2002) 196(4):447–57. doi: 10.1084/jem.20020052 PMC2196057 12186837

[86] Fontenot JD Rasmussen JP Gavin MA Rudensky AY. A function for interleukin 2 in foxp3-expressing regulatory T cells. Nat Immunol (2005) 6(11):1142–51. doi: 10.1038/ni1263 16227984

[87] Furtado GC Curotto de Lafaille MA Kutchukhidze N Lafaille JJ. Interleukin 2 signaling is required for cd4(+) regulatory T cell function. J Exp Med (2002) 196(6):851–7. doi: 10.1084/jem.20020190 PMC2194060 12235217

[88] de la Rosa M Rutz S Dorninger H Scheffold A. Interleukin-2 is essential for cd4+Cd25+ Regulatory T cell function. Eur J Immunol (2004) 34(9):2480–8. doi: 10.1002/eji.200425274 15307180

[89] Ross SH Cantrell DA. Signaling and function of interleukin-2 in T lymphocytes. Annu Rev Immunol (2018) 36:411–33. doi: 10.1146/annurev-immunol-042617-053352 PMC6472684 29677473

[90] Pandiyan P Zheng L Ishihara S Reed J Lenardo MJ. Cd4+Cd25+Foxp3+ Regulatory T cells induce cytokine deprivation-mediated apoptosis of effector cd4+ T cells. Nat Immunol (2007) 8(12):1353–62. doi: 10.1038/ni1536 17982458

[91] Barthlott T Moncrieffe H Veldhoen M Atkins CJ Christensen J O’Garra A. Cd25+ Cd4+ T cells compete with naive cd4+ T cells for IL-2 and exploit it for the induction of IL-10 production. Int Immunol (2005) 17(3):279–88. doi: 10.1093/intimm/dxh207 15684039

[92] Busse D de la Rosa M Hobiger K Thurley K Flossdorf M Scheffold A. Competing feedback loops shape IL-2 signaling between helper and regulatory T lymphocytes in cellular microenvironments. Proc Natl Acad Sci USA (2010) 107(7):3058–63. doi: 10.1073/pnas.0812851107 PMC2840293 20133667

[93] Whiteside TL Jackson EK. Adenosine and prostaglandin E2 production by human inducible regulatory T cells in health and disease. Front Immunol (2013) 4:212. doi: 10.3389/fimmu.2013.00212 23898333 PMC3722515

[94] Kobie JJ Shah PR Yang L Rebhahn JA Fowell DJ Mosmann TR. T regulatory and primed uncommitted cd4 T cells express cd73, which suppresses effector cd4 T cells by converting 5’-adenosine monophosphate to adenosine. J Immunol (2006) 177(10):6780–6. doi: 10.4049/jimmunol.177.10.6780 17082591

[95] Allard B Longhi MS Robson SC Stagg J. The ectonucleotidases cd39 and cd73: novel checkpoint inhibitor targets. Immunol Rev (2017) 276(1):121–44. doi: 10.1111/imr.12528 PMC5338647 28258700

[96] Deaglio S Dwyer KM Gao W Friedman D Usheva A Erat A. Adenosine generation catalyzed by cd39 and cd73 expressed on regulatory T cells mediates immune suppression. J Exp Med (2007) 204(6):1257–65. doi: 10.1084/jem.20062512 PMC2118603 17502665

[97] Bopp T Becker C Klein M Klein-Hessling S Palmetshofer A Serfling E. Cyclic adenosine monophosphate is a key component of regulatory T cell-mediated suppression. J Exp Med (2007) 204(6):1303–10. doi: 10.1084/jem.20062129 PMC2118605 17502663

[98] Klein M Bopp T. Cyclic amp represents a crucial component of Treg cell-mediated immune regulation. Front Immunol (2016) 7:315. doi: 10.3389/fimmu.2016.00315 27621729 PMC5002888

[99] Bodor J Bopp T Vaeth M Klein M Serfling E Hunig T. Cyclic amp underpins suppression by regulatory T cells. Eur J Immunol (2012) 42(6):1375–84. doi: 10.1002/eji.201141578 22678893

[100] Cyster JG Allen CDC. B cell responses: cell interaction dynamics and decisions. Cell (2019) 177(3):524–40. doi: 10.1016/j.cell.2019.03.016 PMC6538279 31002794

[101] Mauri C Ehrenstein MR. The ‘Short’ History of regulatory B cells. Trends Immunol (2008) 29(1):34–40. doi: 10.1016/j.it.2007.10.004 18289504

[102] Matsumura Y Watanabe R Fujimoto M. Suppressive mechanisms of regulatory B cells in mice and humans. Int Immunol (2023) 35(2):55–65. doi: 10.1093/intimm/dxac048 36153768 PMC9918854

[103] Blair PA Norena LY Flores-Borja F Rawlings DJ Isenberg DA Ehrenstein MR. Cd19(+)Cd24(Hi)Cd38(Hi) B cells exhibit regulatory capacity in healthy individuals but are functionally impaired in systemic lupus erythematosus patients. Immunity (2010) 32(1):129–40. doi: 10.1016/j.immuni.2009.11.009 20079667

[104] Iwata Y Matsushita T Horikawa M Dilillo DJ Yanaba K Venturi GM. Characterization of a rare IL-10-competent B-cell subset in humans that parallels mouse regulatory B10 cells. Blood (2011) 117(2):530–41. doi: 10.1182/blood-2010-07-294249 PMC3031478 20962324

[105] Yanaba K Bouaziz JD Haas KM Poe JC Fujimoto M Tedder TF. A regulatory B cell subset with a unique CD1dhiCD5+ Phenotype controls T cell-dependent inflammatory responses. Immunity (2008) 28(5):639–50. doi: 10.1016/j.immuni.2008.03.017 18482568

[106] Wang RX Yu CR Dambuza IM Mahdi RM Dolinska MB Sergeev YV. Interleukin-35 induces regulatory B cells that suppress autoimmune disease. Nat Med (2014) 20(6):633–41. doi: 10.1038/nm.3554 PMC4048323 24743305

[107] Sattler S Ling GS Xu D Hussaarts L Romaine A Zhao H. IL-10 producing regulatory B cells induced by IL-33 (Breg(IL-33)) effectively attenuate mucosal inflammatory responses in the gut. J Autoimmun (2014) 50(100):107–22. doi: 10.1016/j.jaut.2014.01.032 PMC4012142 24491821

[108] Chekol Abebe E Asmamaw Dejenie T Mengie Ayele T Dagnew Baye N Agegnehu Teshome A Tilahun Muche Z. The role of regulatory B cells in health and diseases: A systemic review. J Inflammation Res (2021) 14:75–84. doi: 10.2147/JIR.S286426 PMC7811483 33469337

[109] Das A Ellis G Pallant C Lopes AR Khanna P Peppa D. IL-10 producing regulatory B cells in the pathogenesis of chronic hepatitis B virus infection. J Immunol (2012) 189(8):3925–35. doi: 10.4049/jimmunol.1103139 PMC3480715 22972930

[110] Siewe B Stapleton JT Martinson J Keshavarzian A Kazmi N Demarais PM. Regulatory B cell frequency correlates with markers of HIV disease progression and attenuates anti-HIV cd8(+) T cell function in vitro. J Leukoc Biol (2013) 93(5):811–8. doi: 10.1189/jlb.0912436 PMC3629440 23434518

[111] Gong Y Zhao C Zhao P Wang M Zhou G Han F. Role of IL-10-producing regulatory B cells in chronic hepatitis B virus infection. Dig Dis Sci (2015) 60(5):1308–14. doi: 10.1007/s10620-014-3358-1 25260658

[112] Horikawa M Weimer ET DiLillo DJ Venturi GM Spolski R Leonard WJ. Regulatory B cell (B10 cell) expansion during Listeria infection governs innate and cellular immune responses in mice. J Immunol (2013) 190(3):1158–68. doi: 10.4049/jimmunol.1201427 PMC3552111 23275601

[113] Neves P Lampropoulou V Calderon-Gomez E Roch T Stervbo U Shen P. Signaling via the MyD88 adaptor protein in B cells suppresses protective immunity during *Salmonella typhimurium* infection. Immunity (2010) 33(5):777–90. doi: 10.1016/j.immuni.2010.10.016 21093317

[114] Carter NA Vasconcellos R Rosser EC Tulone C Munoz-Suano A Kamanaka M. Mice lacking endogenous IL-10-producing regulatory B cells develop exacerbated disease and present with an increased frequency of Th1/Th17 but a decrease in regulatory T cells. J Immunol (2011) 186(10):5569–79. doi: 10.4049/jimmunol.1100284 21464089

[115] Flores-Borja F Bosma A Ng D Reddy V Ehrenstein MR Isenberg DA. Cd19+Cd24hicd38hi B cells maintain regulatory T cells while limiting Th1 and Th17 differentiation. Sci Transl Med (2013) 5(173):173ra23. doi: 10.1126/scitranslmed.3005407 23427243

[116] Klinker MW Lundy SK. Multiple mechanisms of immune suppression by B lymphocytes. Mol Med (2012) 18(1):123–37. doi: 10.2119/molmed.2011.00333 PMC3276396 22033729

[117] Ding Q Yeung M Camirand G Zeng Q Akiba H Yagita H. Regulatory B cells are identified by expression of TIM-1 and can be induced through TIM-1 ligation to promote tolerance in mice. J Clin Invest (2011) 121(9):3645–56. doi: 10.1172/JCI46274 PMC3163958 21821911

[118] Yoshizaki A Miyagaki T DiLillo DJ Matsushita T Horikawa M Kountikov EI. Regulatory B cells control T-cell autoimmunity through IL-21-dependent cognate interactions. Nature (2012) 491(7423):264–8. doi: 10.1038/nature11501 PMC3493692 23064231

[119] Mohib K Cherukuri A Zhou Y Ding Q Watkins SC Rothstein DM. Antigen-dependent interactions between regulatory B cells and T cells at the T:B border inhibit subsequent T cell interactions with DCs. Am J Transplant (2020) 20(1):52–63. doi: 10.1111/ajt.15546 31355483 PMC8117747

[120] Ise W Fujii K Shiroguchi K Ito A Kometani K Takeda K. T follicular helper cell-germinal center B cell interaction strength regulates entry into plasma cell or recycling germinal center cell fate. Immunity (2018) 48(4):702–15 e4. doi: 10.1016/j.immuni.2018.03.027 29669250

[121] Khan AR Hams E Floudas A Sparwasser T Weaver CT Fallon PG. PD-L1hi B cells are critical regulators of humoral immunity. Nat Commun (2015) 6:5997. doi: 10.1038/ncomms6997 25609381

[122] Keir ME Butte MJ Freeman GJ Sharpe AH. PD-1 and its ligands in tolerance and immunity. Annu Rev Immunol (2008) 26:677–704. doi: 10.1146/annurev.immunol.26.021607.090331 18173375 PMC10637733

[123] Sun CM Deriaud E Leclerc C Lo-Man R. Upon TLR9 signaling, cd5+ B cells control the IL-12-dependent Th1-priming capacity of neonatal DCs. Immunity (2005) 22(4):467–77. doi: 10.1016/j.immuni.2005.02.008 15845451

[124] Matsumoto M Baba A Yokota T Nishikawa H Ohkawa Y Kayama H. Interleukin-10-producing plasmablasts exert regulatory function in autoimmune inflammation. Immunity (2014) 41(6):1040–51. doi: 10.1016/j.immuni.2014.10.016 25484301

[125] Fillatreau S Sweenie CH McGeachy MJ Gray D Anderton SM. B cells regulate autoimmunity by provision of IL-10. Nat Immunol (2002) 3(10):944–50. doi: 10.1038/ni833 12244307

[126] Carter NA Rosser EC Mauri C. Interleukin-10 produced by B cells is crucial for the suppression of Th1/Th17 responses, induction of T regulatory type 1 cells and reduction of collagen-induced arthritis. Arthritis Res Ther (2012) 14(1):R32. doi: 10.1186/ar3736 22315945 PMC3392827

[127] Ronet C Hauyon-La Torre Y Revaz-Breton M Mastelic B Tacchini-Cottier F Louis J. Regulatory B cells shape the development of Th2 immune responses in BALB/c mice infected with *Leishmania major* through IL-10 production. J Immunol (2010) 184(2):886–94. doi: 10.4049/jimmunol.0901114 19966209

[128] Boldison J Da Rosa LC Davies J Wen L Wong FS. Dendritic cells license regulatory B cells to produce IL-10 and mediate suppression of antigen-specific cd8 T cells. Cell Mol Immunol (2020) 17(8):843–55. doi: 10.1038/s41423-019-0324-z PMC7395736 31728048

[129] Shen P Roch T Lampropoulou V O’Connor RA Stervbo U Hilgenberg E. IL-35-producing B cells are critical regulators of immunity during autoimmune and infectious diseases. Nature (2014) 507(7492):366–70. doi: 10.1038/nature12979 PMC4260166 24572363

[130] Kaku H Cheng KF Al-Abed Y Rothstein TL. A novel mechanism of B cell-mediated immune suppression through cd73 expression and adenosine production. J Immunol (2014) 193(12):5904–13. doi: 10.4049/jimmunol.1400336 PMC4321875 25392527

[131] Saze Z Schuler PJ Hong CS Cheng D Jackson EK Whiteside TL. Adenosine production by human B cells and B cell-mediated suppression of activated T cells. Blood (2013) 122(1):9–18. doi: 10.1182/blood-2013-02-482406 23678003 PMC3701906

[132] Wang L Fu Y Yu B Jiang X Liu H Liu J. Hsp70, a novel regulatory molecule in B cell-mediated suppression of autoimmune diseases. J Mol Biol (2021) 433(1):166634. doi: 10.1016/j.jmb.2020.08.019 32860772

[133] Veglia F Sanseviero E Gabrilovich DI. Myeloid-derived suppressor cells in the era of increasing myeloid cell diversity. Nat Rev Immunol (2021) 21(8):485–98. doi: 10.1038/s41577-020-00490-y PMC7849958 33526920

[134] Dietrich O Heinz A Goldmann O Geffers R Beineke A Hiller K. Dysregulated immunometabolism is associated with the generation of myeloid-derived suppressor cells in *Staphylococcus aureus* chronic infection. J Innate Immun (2022) 14(3):257–74. doi: 10.1159/000519306 PMC9149459 34763332

[135] Bronte V Brandau S Chen SH Colombo MP Frey AB Greten TF. Recommendations for myeloid-derived suppressor cell nomenclature and characterization standards. Nat Commun (2016) 7:12150. doi: 10.1038/ncomms12150 27381735 PMC4935811

[136] Cassetta L Baekkevold ES Brandau S Bujko A Cassatella MA Dorhoi A. Deciphering myeloid-derived suppressor cells: isolation and markers in humans, mice and non-human primates. Cancer Immunol Immunother (2019) 68(4):687–97. doi: 10.1007/s00262-019-02302-2 PMC6447515 30684003

[137] Goldmann O Beineke A Medina E. Identification of a novel subset of myeloid-derived suppressor cells during chronic staphylococcal infection that resembles immature eosinophils. J Infect Dis (2017) 216(11):1444–51. doi: 10.1093/infdis/jix494 29029332

[138] Jaufmann J Lelis FJN Teschner AC Fromm K Rieber N Hartl D. Human monocytic myeloid-derived suppressor cells impair B-cell phenotype and function in vitro. Eur J Immunol (2020) 50(1):33–47. doi: 10.1002/eji.201948240 31557313

[139] Wang Y Schafer CC Hough KP Tousif S Duncan SR Kearney JF. Myeloid-derived suppressor cells impair B cell responses in lung cancer through IL-7 and stat5. J Immunol (2018) 201(1):278–95. doi: 10.4049/jimmunol.1701069 PMC6008229 29752311

[140] Goh CC Roggerson KM Lee HC Golden-Mason L Rosen HR Hahn YS. Hepatitis C virus-induced myeloid-derived suppressor cells suppress NK cell IFN-gamma production by altering cellular metabolism via arginase-1. J Immunol (2016) 196(5):2283–92. doi: 10.4049/jimmunol.1501881 PMC4761460 26826241

[141] Li H Han Y Guo Q Zhang M Cao X. Cancer-expanded myeloid-derived suppressor cells induce anergy of nk cells through membrane-bound TGF-beta 1. J Immunol (2009) 182(1):240–9. doi: 10.4049/jimmunol.182.1.240 19109155

[142] Elkabets M Ribeiro VS Dinarello CA Ostrand-Rosenberg S Di Santo JP Apte RN. IL-1beta regulates a novel myeloid-derived suppressor cell subset that impairs NK cell development and function. Eur J Immunol (2010) 40(12):3347–57. doi: 10.1002/eji.201041037 PMC3373225 21110318

[143] du Plessis N Loebenberg L Kriel M von Groote-Bidlingmaier F Ribechini E Loxton AG. Increased frequency of myeloid-derived suppressor cells during active tuberculosis and after recent *Mycobacterium tuberculosis* infection suppresses T-cell function. Am J Respir Crit Care Med (2013) 188(6):724–32. doi: 10.1164/rccm.201302-0249OC 23885784

[144] Knaul JK Jorg S Oberbeck-Mueller D Heinemann E Scheuermann L Brinkmann V. Lung-residing myeloid-derived suppressors display dual functionality in murine pulmonary tuberculosis. Am J Respir Crit Care Med (2014) 190(9):1053–66. doi: 10.1164/rccm.201405-0828OC 25275852

[145] Tsiganov EN Verbina EM Radaeva TV Sosunov VV Kosmiadi GA Nikitina IY. Gr-1dimcd11b+ Immature myeloid-derived suppressor cells but not neutrophils are markers of lethal tuberculosis infection in mice. J Immunol (2014) 192(10):4718–27. doi: 10.4049/jimmunol.1301365 PMC4537794 24711621

[146] Tebartz C Horst SA Sparwasser T Huehn J Beineke A Peters G. A major role for myeloid-derived suppressor cells and a minor role for regulatory T cells in immunosuppression during *Staphylococcus aureus* infection. J Immunol (2015) 194(3):1100–11. doi: 10.4049/jimmunol.1400196 25548227

[147] Heim CE Vidlak D Scherr TD Kozel JA Holzapfel M Muirhead DE. Myeloid-derived suppressor cells contribute to *Staphylococcus aureus* orthopedic biofilm infection. J Immunol (2014) 192(8):3778–92. doi: 10.4049/jimmunol.1303408 PMC4004612 24646737

[148] Tam JW Kullas AL Mena P Bliska JB van der Velden AW. Cd11b+ Ly6chi ly6g- immature myeloid cells recruited in response to Salmonella enterica serovar Typhimurium infection exhibit protective and immunosuppressive properties. Infect Immun (2014) 82(6):2606–14. doi: 10.1128/IAI.01590-13 PMC4019163 24711563

[149] O’Connor MA Rastad JL Green WR. The role of myeloid-derived suppressor cells in viral infection. Viral Immunol (2017) 30(2):82–97. doi: 10.1089/vim.2016.0125 28051364 PMC5346953

[150] Norris BA Uebelhoer LS Nakaya HI Price AA Grakoui A Pulendran B. Chronic but not acute virus infection induces sustained expansion of myeloid suppressor cell numbers that inhibit viral-specific T cell immunity. Immunity (2013) 38(2):309–21. doi: 10.1016/j.immuni.2012.10.022 PMC3869405 23438822

[151] Tacke RS Lee HC Goh C Courtney J Polyak SJ Rosen HR. Myeloid suppressor cells induced by hepatitis C virus suppress T-cell responses through the production of reactive oxygen species. Hepatology (2012) 55(2):343–53. doi: 10.1002/hep.24700 PMC3358038 21953144

[152] Rosado-Sanchez I De Pablo-Bernal R Rull A Gonzalez J Moreno S Vinuesa D. Increased frequencies of myeloid-derived suppressor cells precede immunodiscordance in HIV-infected subjects. Front Immunol (2020) 11:581307. doi: 10.3389/fimmu.2020.581307 33240269 PMC7677300

[153] Vollbrecht T Stirner R Tufman A Roider J Huber RM Bogner JR. Chronic progressive HIV-1 infection is associated with elevated levels of myeloid-derived suppressor cells. AIDS (2012) 26(12):F31–7. doi: 10.1097/QAD.0b013e328354b43f 22526518

[154] Wang L Zhao J Ren JP Wu XY Morrison ZD Elgazzar MA. Expansion of myeloid-derived suppressor cells promotes differentiation of regulatory T cells in HIV-1+ Individuals. AIDS (2016) 30(10):1521–31. doi: 10.1097/QAD.0000000000001083 PMC4889474 26959508

[155] Park SJ Nam DE Seong HC Hahn YS. New discovery of myeloid-derived suppressor cell’s tale on viral infection and COVID-19. Front Immunol (2022) 13:842535. doi: 10.3389/fimmu.2022.842535 35185933 PMC8850309

[156] Wu G Meininger CJ McNeal CJ Bazer FW Rhoads JM. Role of L-arginine in nitric oxide synthesis and health in humans. Adv Exp Med Biol (2021) 1332:167–87. doi: 10.1007/978-3-030-74180-8_10 34251644

[157] Bronte V Zanovello P. Regulation of immune responses by L-arginine metabolism. Nat Rev Immunol (2005) 5(8):641–54. doi: 10.1038/nri1668 16056256

[158] Morris SM Jr. Arginine metabolism: boundaries of our knowledge. J Nutr (2007) 137(6 Suppl 2):1602S–9S. doi: 10.1093/jn/137.6.1602S 17513435

[159] Rodriguez PC Quiceno DG Zabaleta J Ortiz B Zea AH Piazuelo MB. Arginase I production in the tumor microenvironment by mature myeloid cells inhibits T-cell receptor expression and antigen-specific T-cell responses. Cancer Res (2004) 64(16):5839–49. doi: 10.1158/0008-5472.CAN-04-0465 15313928

[160] Rodriguez PC Hernandez CP Quiceno D Dubinett SM Zabaleta J Ochoa JB. Arginase I in myeloid suppressor cells is induced by COX-2 in lung carcinoma. J Exp Med (2005) 202(7):931–9. doi: 10.1084/jem.20050715 PMC2213169 16186186

[161] Rodriguez PC Quiceno DG Ochoa AC. L-arginine availability regulates T-lymphocyte cell-cycle progression. Blood (2007) 109(4):1568–73. doi: 10.1182/blood-2006-06-031856 PMC1794048 17023580

[162] Rodriguez PC Zea AH Culotta KS Zabaleta J Ochoa JB Ochoa AC. Regulation of T cell receptor cd3zeta chain expression by L-arginine. J Biol Chem (2002) 277(24):21123–9. doi: 10.1074/jbc.M110675200 11950832

[163] Zea AH Rodriguez PC Culotta KS Hernandez CP DeSalvo J Ochoa JB. L-arginine modulates cd3zeta expression and T cell function in activated human T lymphocytes. Cell Immunol (2004) 232(1-2):21–31. doi: 10.1016/j.cellimm.2005.01.004 15922712

[164] Raber PL Thevenot P Sierra R Wyczechowska D Halle D Ramirez ME. Subpopulations of myeloid-derived suppressor cells impair T cell responses through independent nitric oxide-related pathways. Int J Cancer (2014) 134(12):2853–64. doi: 10.1002/ijc.28622 PMC3980009 24259296

[165] Gehad AE Lichtman MK Schmults CD Teague JE Calarese AW Jiang Y. Nitric oxide-producing myeloid-derived suppressor cells inhibit vascular E-selectin expression in human squamous cell carcinomas. J Invest Dermatol (2012) 132(11):2642–51. doi: 10.1038/jid.2012.190 PMC3449043 22718118

[166] Mazzoni A Bronte V Visintin A Spitzer JH Apolloni E Serafini P. Myeloid suppressor lines inhibit T cell responses by an no-dependent mechanism. J Immunol (2002) 168(2):689–95. doi: 10.4049/jimmunol.168.2.689 11777962

[167] Fischer TA Palmetshofer A Gambaryan S Butt E Jassoy C Walter U. Activation of cGMP-dependent protein kinase ibeta inhibits interleukin 2 release and proliferation of T cell receptor-stimulated human peripheral T cells. J Biol Chem (2001) 276(8):5967–74. doi: 10.1074/jbc.M009781200 11073964

[168] Sareila O Kelkka T Pizzolla A Hultqvist M Holmdahl R. NOX2 complex-derived ROS as immune regulators. Antioxid Redox Signal (2011) 15(8):2197–208. doi: 10.1089/ars.2010.3635 20919938

[169] Nagaraj S Gupta K Pisarev V Kinarsky L Sherman S Kang L. Altered recognition of antigen is a mechanism of cd8+ T cell tolerance in cancer. Nat Med (2007) 13(7):828–35. doi: 10.1038/nm1609 PMC2135607 17603493

[170] Beury DW Carter KA Nelson C Sinha P Hanson E Nyandjo M. Myeloid-derived suppressor cell survival and function are regulated by the transcription factor Nrf2. J Immunol (2016) 196(8):3470–8. doi: 10.4049/jimmunol.1501785 PMC4821672 26936880

[171] Yaseen MM Abuharfeil NM Darmani H Daoud A. Mechanisms of immune suppression by myeloid-derived suppressor cells: the role of interleukin-10 as a key immunoregulatory cytokine. Open Biol (2020) 10(9):200111. doi: 10.1098/rsob.200111 32931721 PMC7536076

[172] Hart KM Byrne KT Molloy MJ Usherwood EM Berwin B. IL-10 immunomodulation of myeloid cells regulates a murine model of ovarian cancer. Front Immunol (2011) 2:29. doi: 10.3389/fimmu.2011.00029 22566819 PMC3342001

[173] Wang R Green DR. Metabolic reprogramming and metabolic dependency in T cells. Immunol Rev (2012) 249(1):14–26. doi: 10.1111/j.1600-065X.2012.01155.x 22889212 PMC3422760

[174] van der Windt GJ Pearce EL. Metabolic switching and fuel choice during T-cell differentiation and memory development. Immunol Rev (2012) 249(1):27–42. doi: 10.1111/j.1600-065X.2012.01150.x 22889213 PMC3645891

[175] Lunt SY Vander Heiden MG. Aerobic glycolysis: meeting the metabolic requirements of cell proliferation. Annu Rev Cell Dev Biol (2011) 27:441–64. doi: 10.1146/annurev-cellbio-092910-154237 21985671

[176] Goldmann O Medina E. Myeloid-derived suppressor cells impair cd4+ T cell responses during chronic *Staphylococcus aureus* infection via lactate metabolism. Cell Mol Life Sci (2023) 80(8):221. doi: 10.1007/s00018-023-04875-9 37480485 PMC10363054

